# In Situ Modification of Polyisoprene by Organo-Nanoclay during Emulsion Polymerization for Reinforcing Natural Rubber Thin Films

**DOI:** 10.3390/polym11081338

**Published:** 2019-08-12

**Authors:** Jadsadaporn Chouytan, Ekwipoo Kalkornsurapranee, Christopher M. Fellows, Wisut Kaewsakul

**Affiliations:** 1 Department of Materials Science and Technology, Faculty of Science, Prince of Songkla University, Hat Yai Campus, Songkhla 90110, Thailand; 2 School of Science and Technology, University of New England, Armidale, NSW 2351, Australia; 3 Elastomer Technology and Engineering, Department of Mechanics of Solids, Surfaces and Systems, Faculty of Engineering Technology, University of Twente, 7522 NB Enschede, The Netherlands

**Keywords:** nanocomposite, elastomer, latex, filler, reinforcement

## Abstract

Nanoclay-modified polyisoprene latexes were prepared and then used as a reinforcing component in natural rubber (NR) thin films. Starve-fed emulsion (SFE) polymerization gives a higher conversion than the batch emulsion (BE), while the gel and coagulation contents from both systems are comparable. This is attributed to the SFE that provides a smaller average polymer particle size which in turn results in a greater polymerization locus, promoting the reaction rate. The addition of organo-nanoclay during synthesizing polyisoprene significantly lessens the polymerization efficiency because the nanoclay has a potential to suppress nucleation process of the reaction. It also intervenes the stabilizing efficiency of the surfactant—SDS or sodium dodecyl sulfate, giving enlarged average sizes of the polymer particles suspended in the latexes. TEM images show that nanoclay particles are attached on and/or inserted in the polymer particles. XRD and thermal (differential scanning calorimetry (DSC) and thermogravimetric analysis (TGA)) analyses were employed to assess the d-spacing of nanoclay structure in NR nanocomposite films, respectively. Based on the overall results, 5 wt% of nanoclay relative to the monomer content utilized to alter the polyisoprene during emulsion polymerization is an optimum amount since the silicate plates of nanoclay in the composite exhibit the largest d-spacing which maximizes the extent of immobilized polymer constituent, giving the highest mechanical properties to the films. The excessive amounts of nanoclay used, i.e., 7 and 10 wt% relative to the monomer content, reduce the reinforcing power because of the re-agglomeration effect.

## 1. Introduction

Thin film-based natural rubber (NR) products are typically fabricated from NR latex and other compounds. Condoms and gloves are good examples of products in this category [1,2]. One of the crucial end-use properties of these products, which is highly required by the users and a selling point of these products, is the film thickness [3]. In general, users prefer these rubber film products as thin as possible since touch sensitivity is better while wearing a thinner film [4]. However, reducing a thickness of the rubber films is usually accompanied by a decrease in the mechanical properties [5]. Thus, a proper trade-off between these aspects of the thin film products needs to be fine-tuned.

To reinforce the thin films based on elastomer latexes, the conventional reinforcing fillers that are commonly employed for solid rubbers, e.g., tires, high mechanical goods, etc., are not feasible. These fillers require a high mechanical shear rate during mixing them with a rubber. With films applied under low-shear conditions, the particles of reinforcing fillers are mostly in the structure of agglomerates or clusters [6,7]. Basically, the reinforcing power of filler is significantly enhanced by breaking these clusters to increase the surface area of the filler particles dispersed in the rubber matrix, giving an increased filler–rubber interaction [8]. Hence, a sufficient high shearing action during mixing is necessary to develop this interfacial interaction, contributing to the preferable reinforcement level [9,10]. For solid rubbers, the mixing is commonly carried out in a high-shear mixer that has an adequate power to break and disperse the filler particles [11,12]. However, compounding a rubber with additives in the latex state needs to be done under a rather low stirring speed or shearing action which is insufficient to break the filler particles. Too high a speed of stirring during latex compounding causes undesired coagulation of the latex because of the inherent instability of colloidal systems. Therefore, conventional reinforcing fillers are not applicable in this case [13,14].

Nanofiller in a colloidal or dispersion form which may fulfill the requirements to effectively reinforce rubber thin films based on latexes. Fumed silica has been used to prepare a nanosilica dispersion for reinforcing NR using water as a medium [15,16]. This nanosilica dispersion did not require high shear rate to break the particles during compounding since it was already composed of dispersed nanoscale particles (50–200 nm). Hence, it can easily be mixed with the compounds in latex state and gives high reinforcement to rubber thin films [17]. However, this colloidal nanosilica is rather expensive which in turn increases the production costs. Therefore, the alternatives to colloidal nanosilicas, which provide comparable or superior reinforcing property while offering much lower price, can be highly beneficial to the latex-based manufacturing industry. Even though commercially acceptable prices of thinner condoms or gloves can be two to three times higher than the normal film products [18], any attempt to reduce the production costs is attractive to the manufacturers. 

In order to make nanofillers well-dispersed in a rubber matrix, the researchers in this field have paid close attention to many aspects of the particular composites. These include: studies concerning intrinsic properties of raw materials in the composites, e.g., the modifications of polymers [19,20], fillers [21,22], and processing aids [23,24]; novel syntheses of key materials for the composites [25,26,27]; and the optimizations of different processing techniques and conditions, e.g., melt [28,29] and solution [30,31] mixing, mixing time and temperature, mixing procedure, pre-dispersion methods, etc., [32,33]. This technology remains challenging since the properties of a composite depend strongly on the processing conditions as well as composition. 

In situ modification of polymers by nanofillers during polymerization is recently introduced [34]. The obtained polymers containing nanofillers can be used either as a raw material or as an additive to reinforce other polymers [35,36]. Several techniques have been applied to synthesize these modified polymers, for instance, emulsion [37] and solution [38] polymerizations. Polyisoprene (PIP) has been used as a base polymer for this objective [39]. Boonchoo et al. (2014) [40] conducted the in situ modification of PIP by montmorillonite modified with a quaternary ammonium salt via micro-emulsion polymerization. The latexes were then employed to reinforce the NR thin films. The results showed that the nanoclay-modified PIP latexes can improve the mechanical strength of the NR thin films. However, some crucial aspects of this topic have not yet been reported, e.g., the effect of different emulsion polymerization techniques, the impact of variation of nanoclay content on the modified PIP latexes, and investigation of the reinforcing mechanism of this system. 

The aim of the present work is to synthesize PIP latexes modified with the nanoclay Cloisite 15 (Clay-PIP) and utilize them as reinforcing agents for NR thin films. Two different emulsion polymerizations, i.e., batch and starve-fed techniques, were used to prepare the Clay-PIP latexes. The efficacy of the two techniques was evaluated and the influence of nanoclay content on polymerization efficiency, latex properties, and reinforcement capability in the NR thin films was studied. Basic properties of the obtained latexes investigated were gel and coagulation content, product conversion, particle size, and morphological properties. Thermal properties of the Clay-PIP solids were further analyzed to assess the immobilized polymer in the film nanocomposites. Finally, the ability of the Clay-PIP latexes to reinforce NR thin films was evaluated. 

## 2. Experimental

### 2.1. Materials

Polyisoprene (PIP) latexes were polymerized using the following ingredients: isoprene monomer (analytical research or AR grade, Sigma-Aldrich, Saint Louis, MO, USA); sodium hydroxide solution (RCI Labscan, Bangkok, Thailand) as a neutralizer to remove the inhibitor 4-tert-butylcatechol in the isoprene monomer; anhydrous sodium sulfate as a moisture absorber after the inhibitor removal; organo-nanoclay Cloisite 15A modified with 43 wt% of dimethyl dehydrogenated tallow quaternary ammonium and dry particle size (90 vol%) of less than 13 µm (BYK Additives & Instruments, Wesel, Germany, supplied by Colossal International, Bangkok, Thailand) as a reinforcing nanofiller used to modify polymer particles in the latexes; potassium persulfate or KPS as a thermal initiator; sodium dodecyl sulfate (SDS) (lab research or LR grade) as a primary surfactant; sodium bicarbonate (NaHCO_3_) (AR grade) as a buffer to control emulsion pH; *n*-dodecyl mercaptan (*n*-DM) (LR grade, Aldrich, USA) as a chain transfer agent to control the molecular weight distribution; ethyl methyl ketone (MEK) (LR grade) as a coagulating agent; methanol (Commercial grade, the Excise Department of Thailand) used for washing the coagulated polymer; toluene (AR grade, Fisher Scientific, Loughborough, UK) as a gel extraction reagent; and deionized water (DI, in-house preparation) as an aqueous phase of the polymerization reaction. All chemicals were purchased from Ajax Finechem, NSW, Australia, unless otherwise stated. 

The ingredients used for the preparation of natural rubber thin films reinforced with nanoclay-modified PIP were: high ammonia grade of natural rubber latex concentrate (HA latex) with about 60 wt% dry rubber content or DRC and 0.6 wt% ammonia (Had Sin Rubber, Songkhla, Thailand); sulfur as a vulcanizing agent (Siam Chemicals, Bangkok, Thailand); activator zinc oxide or (ZnO) (Univentures, Bangkok, Thailand); zinc diethyldithiocarbamate (ZDEC) (Performance Additives, Subang Jaya, Malaysia); zinc 2-mercaptobenzothiazole (ZMBT) (Polymer Innovation, Nonthaburi, Thailand), both were used as accelerators; 2, 2′-methylene bis-(6-t-butyl-p-cresol) as an antioxidant (SI group, Schenectady, NY, USA); potassium hydroxide (KOH) (Unid, Seoul, Korea); and potassium laurate (K-Laurate) (Hangzhou Dayangchem, Hangzhou, China) as a surfactant. 

### 2.2. Synthesis of the Nanoclay-Modified Polyisoprene Latexes 

Two different techniques were used for preparing the nanoclay-modified polyisoprene latexes: batch and starve-fed emulsion. An autoclave high-pressure reactor (Well & J Technology, Shenzhen, China) was employed for this synthesis. It consists of a steel jacket inserted with a Teflon inner chamber possessing a capacity of 300 mL, rated to withstand a maximum pressure of 6.0 MPa and a maximum temperature of 250 °C; it is coupled with a pressure gauge as well as inlet and outlet valves. 

#### 2.2.1. Starve-Fed Emulsion Polymerization

The isoprene monomer was fixed at 10 wt% relative to the total amount of the obtainable polymer latex (i.e., 200 g); it was divided into two parts, ¼ and ¾ by weight. The ¼ portion of the monomer was designed to be a part generating the initial micelles for the polymerization reaction [41,42]. It was first mixed with varied amounts of the nanoclay according to the formulations illustrated in Table 1 and then homogenized via a sonicator (Model 275TA, Crest Ultrasonic, Penang, Malaysia) with a frequency of 60 Hz for 30 min at low temperatures of ca. 1–3 °C to prevent evaporation of the isoprene monomer. The obtained suspension was introduced into a flask containing a mixed solution of SDS, *n*-DM, NaHCO_3_, and deionized water. The mixture was stirred for 30 min and sonicated again at a frequency of 60 Hz for 30 min. The obtained mixture became emulsified and was then introduced into a high-pressure reactor. Then, KPS solution was added at once. The reactor was purged with nitrogen gas through the inlet for 5 min and then tightly sealed. The mixture was heated up to 70 °C while stirring with a magnetic bar at 150 rpm. After that, the other ¾ portion of the isoprene monomer was fed into the reactor via a peristaltic pump with a feeding rate of 1 mL/min. Then, the polymerization proceeded for 12 h prior to the isolation of a product.

#### 2.2.2. Batch Emulsion Polymerization

5.0 wt% of nanoclay relative to the isoprene monomer content was mixed with the entire amount of the monomer and homogenized using a sonicator at a frequency of 60 Hz for 30 min at low temperatures of about 1–3 °C. The nanoclay-suspended isoprene monomer was mixed with the same aqueous solution of the SFE system. Homogenization of the mixture was again done by sonication following the same procedure of the SFE method. Then, KPS solution was added. Polymerization was performed at 70 °C under a nitrogen atmosphere for 12 h. The obtainable polymer latexes were kept in sealed brown containers to exclude air and light before analysis of their properties.

### 2.3. Latex Compounding

The unmodified and nanoclay-modified PIP latexes obtained from this synthesis were blended with natural rubber concentrated latex (HA or high ammonia grade, Had Sin Rubber, Thailand) at 10/90 by dry weight of the Clay-PIP/NR in order to fabricate rubber thin films containing variable amounts of the organo-nanoclay. The compound formulation is shown in Table 2. The blended latex was stirred in a container at 25 rpm for 2 h. Then, the surfactants KOH and K-Laurate were added and stirred for 10 min. After that, the other ingredients were charged into the container, and continually stirred for 48 h. This step is commonly known as prevulcanization and is carried out to improve processing properties which in turn lead to optimized final properties of the films [5]. The resulting material is called prevulcanized latex. 

### 2.4. Preparation of the Natural Rubber Thin Films

Rubber thin films were prepared by casting. A certain amount of a latex compound was poured onto a glass plate, followed by manually casting using a metal thickness-controlling bar at a constant speed, and subsequently held for 30 s. The glass plate with a layer of the rubber film deposited was then placed in a hot-air oven at 120 °C for 15 min to vulcanize the film. After that, it was removed from the glass plate by applying talcum powder as an anti-tacking agent on the film surface. The final films had a thickness in the range of 0.03–0.05 mm; they were kept in a dry container prior to measuring their physical and mechanical properties. The properties of the casted films were determined in order to evaluate the reinforcement efficiency of the nanoclay-modified PIP latexes.

### 2.5. Characterizations of the Clay-PIP Latexes 

#### 2.5.1. Conversion, Coagulation, and Gel Content

The latexes were filtered using a 300 mesh steel wire cloth. The residual retained on the steel wire cloth is considered as the coagulum; it was dried and weighed (W_1_). The coagulation percentage can be calculated following Equation (1).

Coagulation (%) = W_1_/W_M_ × 100 (1)

The percentage of conversion was assessed by precipitation of the obtained latexes. A total of 10 g of the latex was precipitated with methyl ethyl ketone (MEK), then washed with methanol and dried to a constant weight (W_2_). The conversion can be calculated using Equation (2).

Conversion (%) = W_2_/W_M_ × W_L_/10 (2)

Gel content of the synthesized polymer latexes can be measured by Soxhlet extraction of the precipitated polymer using toluene as a solvent. The extraction process was performed at the boiling temperature of toluene for 24 h. The residual in a filtering paper was dried until a constant weight (W_3_). The gel content was calculated following Equation (3).

Gel content (%) = W_3_/W_2_ × 100 (3)

where W_1_, W_2_, W_3_, W_M_, and W_L_ are the weights of dried residual coagulum, dried polymer, dried residual after Soxhlet extraction, the initial weight of monomer, and the final weight of the latex obtained, respectively.

#### 2.5.2. Morphology

Transmission electron microscopy (TEM) (Model JEM-2010, JEOL, Peabody, MA, USA) was utilized to image the polymer particles and the nanoclay dispersed in the synthesized latexes. The JEM-2010 has a standard LaB_6_ (Lanthanum hexaboride) filament generating the electron beam which transmits through an ultrathin specimen onto a fluorescent screen as an imaging device operated using a voltage of 160 kV. A latex sample was diluted with DI water at 400 wt% relative to the sample quantity. After that, 10 mL of the diluted latex was passed through a staining process using 10 µL of 0.1 M osmium tetraoxide (OsO_4_) as a dye. The sample was kept in a dark container for 24 h prior to performing the TEM analysis.

#### 2.5.3. Clay Nanostructure Analysis

X-ray diffraction (XRD) (Model PW1050, Philips, Andover, MA, USA) analysis with a Rigaku D/Max II X-ray diffractometer and Cu K-alpha radiation (1.54 Å) generated at 25 mA and 45 kV was used to determine the basal d-spacing. 

#### 2.5.4. Particle Size and Particle Size Distribution

Laser particle size analyzer (LPSA) (COULTER LS230, Jinan Winner Particle Instrument Stock, Jinan, China) was used to determine the particle size and particle size distribution of polymer particles in latexes. A tungsten-halogen lamp was used as a laser light source with a polarization intensity differential scattering detector, scanned from 40 to 2000 µm at a scattering angle of 90°. 

#### 2.5.5. Mechanical Properties

Tensile tester (Model Z005, ZwickRoell, Kennesaw, GA, USA) was used to measure the mechanical properties of the NR thin films filled with nanoclay-modified PIPs. The test protocol was in accordance with ASTM D412 operating with a 100 N load cell and a crosshead speed of 500 mm/min. The samples were prepared using a dumbbell die cut type C. Five samples from each compound were tested and the median stress–strain curves are reported. 

#### 2.5.6. Thermal Analyses

Thermal behaviors of the nanoclay-modified polyisoprene were analyzed using differential scanning calorimetry (DSC 2920, TA Instruments, New Castle, DE, USA) and thermogravimetric analysis (HI-Res TGA 2950, TA Instruments, USA). For DSC analysis, a sample (ca. 5 mg) was cooled down to −100 °C with a cooling rate of 10 °C/min. The sample was then heated from −100 to 50 °C with a heating rate of 5 °C/min under a nitrogen atmosphere. Glass transition temperatures (T_g_) and heat capacity (ΔC_p_) at the transition region from glassy to rubbery state were determined.

Thermogravimetric analysis was carried out over the temperature range 30 to 1000 °C. Approximately 5 mg of sample was heated at a rate of 10 °C/min under nitrogen to evaluate the effect of nanoclay content on thermal stability of the nanoclay-modified PIP.

## 3. Results and Discussion

In the present work, polyisoprene PIP latexes were modified in situ with organo-nanoclay during emulsion polymerization in order to prepare a reinforcing agent for natural rubber thin films. The synthesized nanoclay-modified polyisoprene (Clay-PIP) latexes require appropriate colloidal stability with nanoclay particles being well-dispersed and inserted in or attached on the polymer particles. This work demonstrates how the Clay-PIP latexes were developed. After that, the reinforcing efficiency of the modified latexes in natural rubber (NR) thin films was investigated. 

### 3.1. Synthesis of the Nanoclay-Modified Polyisoprene Latexes

#### 3.1.1. Effect of Different Emulsion Polymerization Techniques

**Figure 1:**
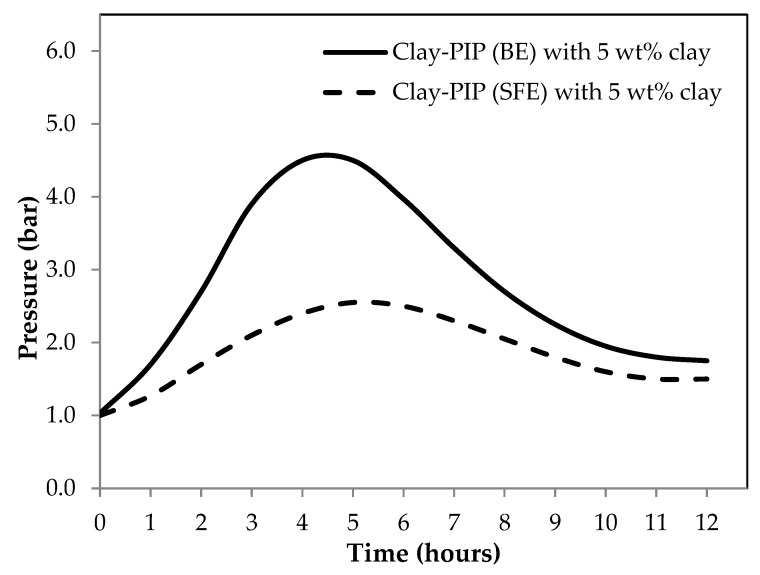
Real-time monitoring data of the internal reactor pressure as a function of reaction time of synthesizing Clay-PIP latexes through batch (BE) and starve-fed (SFE) emulsion polymerization techniques. The organo-nanoclay at 5 wt% relative to the monomer content was employed.

Two emulsion polymerization techniques: batch (BE) and starve-fed (SFE) emulsion polymerization, were utilized to synthesize the nanoclay-modified polyisoprene latexes. The results shown in Table 3 illustrates that these two techniques give similar final properties of the latexes when modified with the same amount of nanoclay, i.e., 5 wt% relative to the monomer content. The BE gives a slightly lower conversion compared to that obtained from the SFE technique, i.e., 65.4 ± 5.9 and 69.9 ± 5.1 wt%, respectively. The internal pressure inside the reactor of SFE shows significantly lower than that of the BE for the whole period of the synthesis: see Figure 1. The difference between them becomes smaller over the synthesizing time since the monomer vapors were circulated into the medium and then participated in the polymerization reaction, finally converting into the polymer chains.

**Figure 2:**
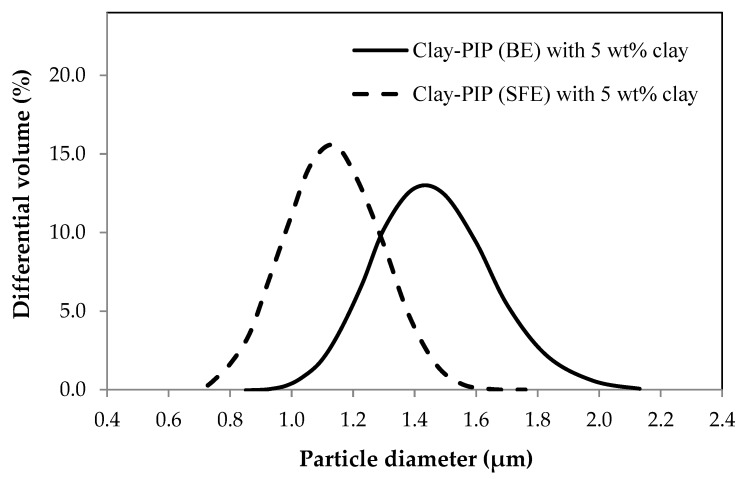
The effect of different emulsion polymerization methods batch (BE) and starve-fed (SFE) techniques, on the particle size of the polyisoprene latexes modified with 5 wt% nanoclay relative to the monomer content.

The SFE system generates a lower internal pressure inside the reactor because the amount of monomer concentration at the initial stage of polymerization was lower than that of the BE. The monomer of SFE system was continually fed into a reactor slowly with a precisely controlled amount of monomer. Hence, that is why this technique is called “starve-fed emulsion polymerization.” The fed monomer will participate rapidly in the on-going polymerization reaction due to the monomer being the limiting reagent, which in turn minimizes the monomer vapors inside the reactor and makes the polymerization more efficient when compared to the BE system. The internal pressure of the reactor and the conversion of obtainable polymers show a good agreement with the results from particle size analysis as displayed in Figure 2 and Table 4.

**Figure 3:**
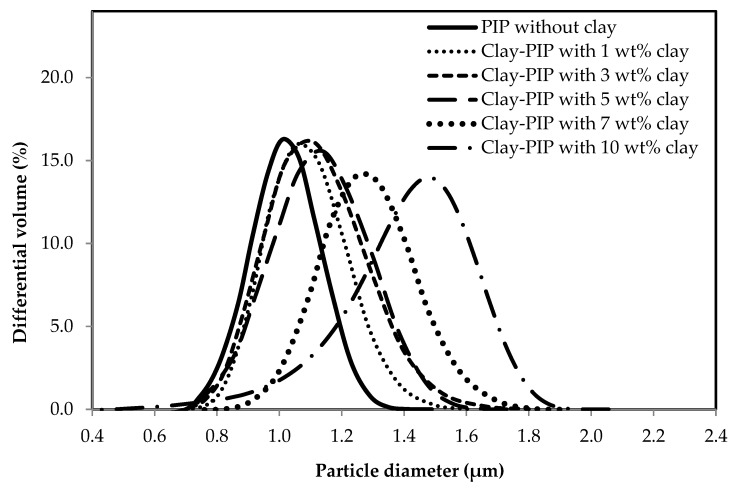
The effect of nanoclay content on the particle size of the nanoclay-modified polyisoprene latexes prepared using SFE polymerization.

In general, the polymer particle size from a typical emulsion polymerization falls in the range of 50–500 nm [3]. However, the synthesizing recipes in this study contain a rather small amount of surfactant, i.e., 0.25 wt%, moreover they have an organo-nanoclay involved during the polymerization which induces more flocculation of polymer particles, leading to enlarged polymer particle sizes as evidenced in Figure 2 and Figure 3. Based on the particle size analysis: see Figure 2, the SFE system gives an average size of the polymer particles smaller than that of the latex derived from BE polymerization. This is because the feeding sequences of the monomer into a reactor of the two systems are different as mentioned earlier (Figure 1). SFE technique feeds the monomer slowly during the polymerization, while the BE starts its polymerization with the entire monomer from the initial stage, leading to more coagulation at a given stirring speed when compared to the SFE system and a smaller number of stable particles at the end of the nucleation stage. 

The smaller the particle size, the higher the particle number concentration (N_P_) for the same mass, leading to more reactive sites in the system due to the greater number of monomer micelles, and thus higher reactivity toward polymerization reaction. A terminology commonly used to refer to the reactive sites is “reaction loci” [43]. The SFE technique appears to have more polymerization loci since it shows a greater N_P_ compared to the BE system. According to the calculation [43] based on the data from this particle size analysis, the N_P_ of the latexes are shown in Table 4. The result indicates that the SFE has a faster rate of polymerization due to more reaction loci resulting in a higher percentage of polymer conversion: see Table 3.

#### 3.1.2. Variation of Nanoclay Contents in the Clay-PIP Latexes 

Based on the results shown in Table 3, adding nanoclay results in a significant decrease in the product conversion. The control sample which was prepared without adding nanoclay has the highest product conversion of 91.7 wt%. Adding nanoclay at 1.0 wt% relative to the monomer amount leads to a considerable drop in the conversion to 78.3 wt%, with further addition of nanoclay from 3.0 to 10.0 wt%, the conversions gradually decrease from 77.8 to 63.4 wt%. This implies that the nanoclay incorporated during the emulsion polymerization of isoprene latexes has an enormous impact on the polymerization efficiency. The addition of organo-nanoclay appears to intervene the stabilizing ability of SDS surfactant toward isoprene micelles. The hydrophobic side of nanoclay can adsorb the SDS as a consequence if less surfactant is available to stabilize the polymer particles. Thus, the latexes tend to have more aggregations and larger particle size. It is commonly known that the concentration of surfactant or stabilizer strongly influences the polymer particle size and particle size distribution. Hence, when the SDS surfactant exhibits low affinity toward isoprene micelles, it can result in an increased interfacial tension between them that causes larger average sizes of polymer particles as illustrated in Figure 3. Consequently, the polymerization loci are reduced, and so the decreased conversion. Regarding an inferior shielding efficiency of the surfactant, the rubber particles appear to aggregate, which leads to an increase in polymer particle sizes as well as broadened particle size distributions. This corresponds to the morphology of polymer particles in the resulting latexes as is discussed in the following section. Furthermore, it can be attributed to the fact that nanoclay causes a physical barrier toward the diffusion ability of intermediate monomer species from a droplet to micelles as a consequence in the decreased polymer conversion [44].

Considering the outcome from the calculation of polymer particle concentration, the latexes prepared with SFE technique without nanoclay, as well as with nanoclay are shown in Table 4. This clearly confirms that an increase in nanoclay content significantly decreases the polymer particle concentration of the resulting latexes. With this circumstance, the system which has a higher polymer particle concentration, i.e., the one with a higher nanoclay content, exhibits lower polymerization loci leading to the reduced product conversion (see Table 3). 

The latexes with higher nanoclay contents also tended to have a gradual increase in the coagulum content (see Table 3). The inclusion of 1–5 wt% of nanoclay relative to the monomer content gives a smaller particle size with a narrower particle size distribution compared to the ones with 7 and 10 wt% nanoclay of which they show a significant increase in the particle size with a broader particle size distribution. A broader particle size distribution is mainly caused by the presence of organo-nanoclay that can reduce stability and induce flocculation of polymer particles. Excessive amounts of the organo-nanoclay cause less shielding capability of the SDS surfactant toward monomer micelles as mentioned earlier, contributing to the aggregation tendency.

#### 3.1.3. Morphology of the Obtained Clay-PIP Latexes 

**Figure 4:**
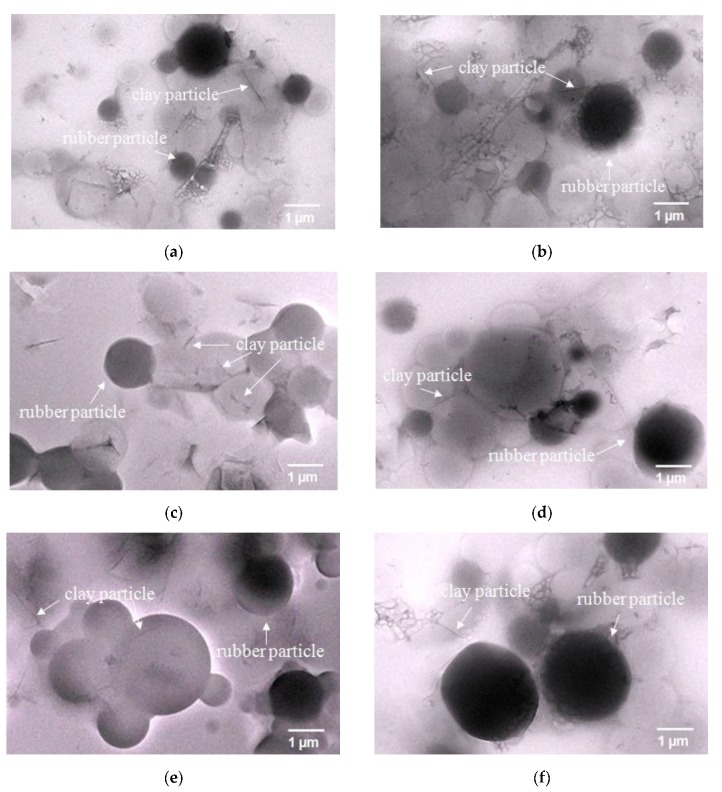
TEM images of nanoclay-modified isoprene latexes prepared through starve-fed emulsion (SFE) polymerization with the variation of nanoclay content: (**a**) 1 wt%; (**b**) 3 wt%; (**c**) 5 wt%; (**d**) 7 wt%; and (**e**) 10 wt% of nanoclay relative to the amount of monomer. The image (**f**) belongs to the latex prepared with batch emulsion (BE) system with 5 wt% of nanoclay relative to the monomer content.

It is obvious from the different product conversions that the particle concentration during emulsion polymerization significantly influences the final properties of the latexes. To confirm this elucidation, a TEM technique was employed to visualize the actual polymer particles and the nanoclay particles distributed in the colloidal latexes. Figure 4 clearly shows that the synthesis technique and the nanoclay content play a role on the morphology of polymer particles. The TEM images of the latexes synthesized with the SFE technique (Figure 4a–e), show that there are nanoclay particles inserted into and/or attached onto the PIP particles, while the BE system (Figure 4f) gives polymer particles with the nanoclay attached onto them, often known as “nanoclay-armored particles” [45].

As aforementioned, the initial polymer particle sizes of a typical emulsion polymerization should be very small compared to the dimensions of the clay particles. However, the present system contains only a small amount of SDS surfactant; therefore, the polymer particles exhibit reduced colloidal stability. In addition, as less amount of the hydrophobe was used the clay particles and the polymer particles tend to aggregate, after the polymer particles have grown to a certain size leading to the insertion of nonoclay particles into the clusters of polymer particles. Furthermore, it can be ascribed that the SFE system has a faster reaction rate as discussed in Figure 2. Additionally, the monomer plus nanoclay was fed into a reactor by continually dispensing a small amount of monomer droplets, so that the nanoclay particles can be trapped in the cluster of polymer droplets during the aggregation in which the polymerization reaction occurs more rapidly than that of the BE system as a consequence in a better holding of the nanoclay. On the other hand, the BE system possesses a slower polymerization rate and the entire amount of monomer plus nanoclay was charged into the reactor from the initial stage. The nanoclay particles might be more likely to lose their holding around the rubber particle cloud due to gravity and agitation effects. Hence, more amounts of nanoclay from the monomer phase are liberated into the water medium. And, some parts of them migrate to the surface of the polymer particles due to high interphase tension between the monomer and nanoclay. 

The variation of nanoclay amount also influences the morphology of polymer particles significantly. In general, the plate shape of nanoclay particles has d-spacing of about 3.63 nm and lateral dimensions in the range 0.2–1.0 μm [44]. All synthesized latexes show their average polymer particle sizes sufficient to encapsulate the nanoclay particles. The TEM images confirm that, when increasing the amounts of nanoclay, the polymer particles tend to have higher aggregation and thus broader particle size distribution. This is due to less shielding efficacy of the surfactant as discussed earlier.

**Figure 5:**
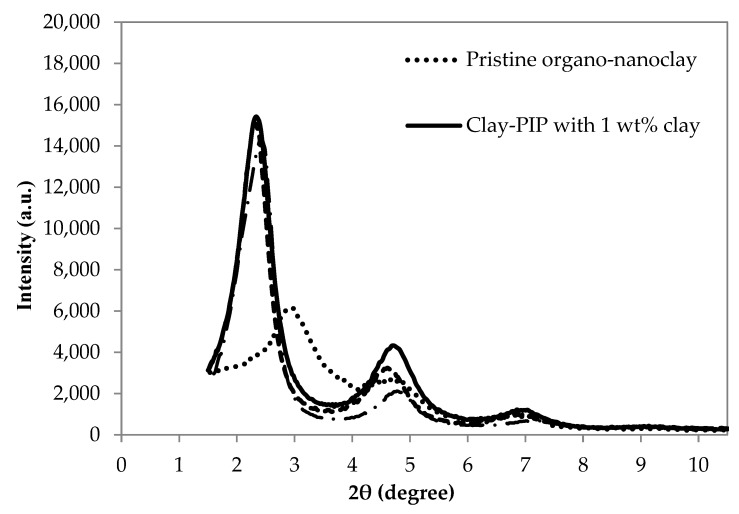
XRD patterns of pristine organo-nanoclay and solid composites based on the Clay-PIP latexes with varied amounts of nanoclay.

To investigate the degree of dispersion of nanoclay within the PIP latex, an XRD technique was utilized. Figure 5 shows the XRD patterns of the pristine organo-nanoclay and the composites based on the Clay-PIP with varied amounts of such nanoclay. The XRD patterns of the pristine nanoclay and the Clay-PIP solids show two important intensity peaks at the 2θ in the range of 2.3–3.1 (larger peak height) and 4.2–5.4 (smaller peak height). These peaks are assigned to the basal spacing of d_001_ and d_002_, respectively. The basal spacing of d_001_ is considered as an indicator of the interlayer distance between the silicate crystal plates of the nanoclay structure that can be calculated following the Bragg’s equation [46]. 

According to the results based on calculation, the d-spacing values of pure nanoclay and polyisoprene solids modified with 1, 5, and 10 wt% of nanoclay relative to the monomer content are 3.63, 3.77, 3.81, and 3.54 nm, respectively. It is clear that the incorporation of nanoclay into the polyisoprene as low as 1 wt% the d-spacing is increased when compared to that of the pristine organo-nanoclay. Adding 5 wt% of nanoclay has shown to be an optimum amount since it gives the maximum d-spacing of the silicate plates. After adding the nanoclay beyond the optimum level, in this case at 10 wt%, the d-spacing of nanoclay tends to reduce, attributed to the overloading of nanoclay and thus a closer distance between the silicate plates. This phenomenon is often ascribed to the effect of reagglomeration of nanoclays [25,47,48]. As expected, the organo-nanoclay is, to some extent, compatible with the isoprene monomer. Utilizing the sonication process, results in an enlarged d-spacing of the silicate layers, giving the desired structure of nanoclay in the polymer matrix, often known as “intercalation and exfoliation” dispersion [49]. These dispersion structures are required since the nanoclay exhibits high power in reinforcing polymeric composites [50].

### 3.2. Use of the Clay-PIP Latexes as a Reinforcing Agent for NR Latex Compounds

#### 3.2.1. Mechanical Properties of the NR Thin Films Reinforced with Clay-PIP

**Figure 6:**
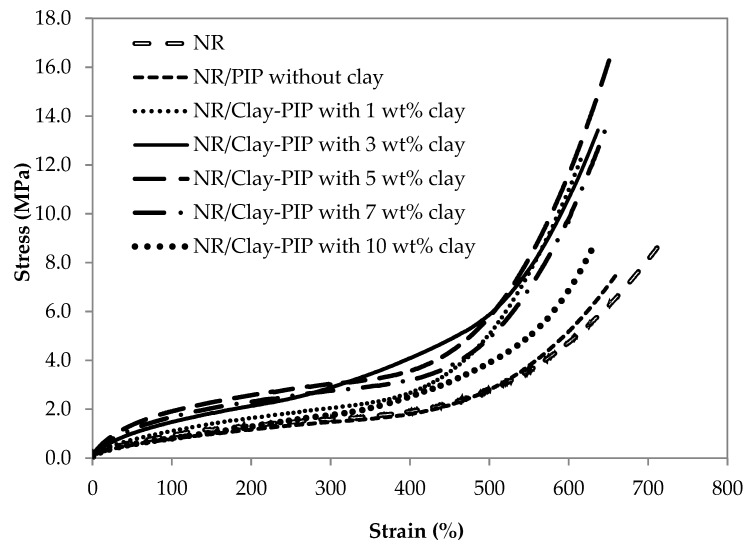
The effect of nanoclay content in the Clay-PIPs on stress–strain behavior of the NR thin films blended with the Clay-PIPs. Each curve was taken based on the median value among five test specimens. The clay contents are relative to the monomer amount.

Variable amounts of nanoclay in the modified PIP latexes differently influence the dispersion and distribution degree of nanofiller added as well as the interaction between nanofiller and polymer, often known as “in-rubber structure” or “bound rubber constituent” [51]. Figure 6 illustrates that the addition of Clay-PIP latexes into NR latex compounds has a significant positive impact on the mechanical properties of the NR thin films. The NR films filled with modified PIP latexes containing organo-nanoclay from 1 to 5 wt% relative to the monomer content show an increase in tensile strength and modulus with a slightly reduced elongation at break. However, adding the modified isoprene latexes containing nanoclay at 7 and 10 wt% leads to reduced strength and modulus of NR thin films, while maintaining elongation at break. The Clay-PIP latexes containing 1 to 5 wt% nanoclay have shown to be able to reinforce NR thin films since they contain the organo-nanoclay predispersed in the PIP latexes, giving a larger d-spacing of the silicate plates (Figure 5). Thus, the interaction between the polymer and nanoclay can be enhanced, leading to a better reinforcement of the natural rubber thin films. Further addition of the nanoclay to the Clay-PIP, i.e., 7 and 10 wt%, gives an adverse effect on the film strength, attributed to the excessive amount of nanoclay causing reagglomeration of the nanoclay as evidenced by the XRD analysis that shows a reduced d-spacing of nanoclay structure (Figure 5).

#### 3.2.2. Indication of Immobilized Polymer in the Composites

The immobilized polymer constituent which is the “bound layer” of polymer molecules on the surface of nanofiller particles is one of the key factors altering the glass transition temperature T_g_ of polymer composites [52]. This filler–polymer interaction causes the bound polymer molecules to be restricted and immobilizable. An increase of the immobilized fraction of the polymer layer surrounding filler particles leads to a shift of the T_g_ to a higher temperature since the polymer molecules are more constrained or in the other words less flexible. Reinforcement of polymers by a particulate filler requires this immobilized polymer structure to be present because the filler and the polymer phases become more compatible due to better intermolecular interfacial interactions. When the reinforced composites undergo a high loading condition, they can withstand the forces better because of low energy dissipation throughout the material [40]—the increase in mechanical strength and modulus (Figure 6). As the immobilized polymer or in-rubber structure is an essential constituent which determines the reinforcing efficiency of polymeric composites, DSC and TGA techniques were used to analyze this structure in order to elucidate the reinforcing mechanism of the rubber films in this study.

**Figure 7:**
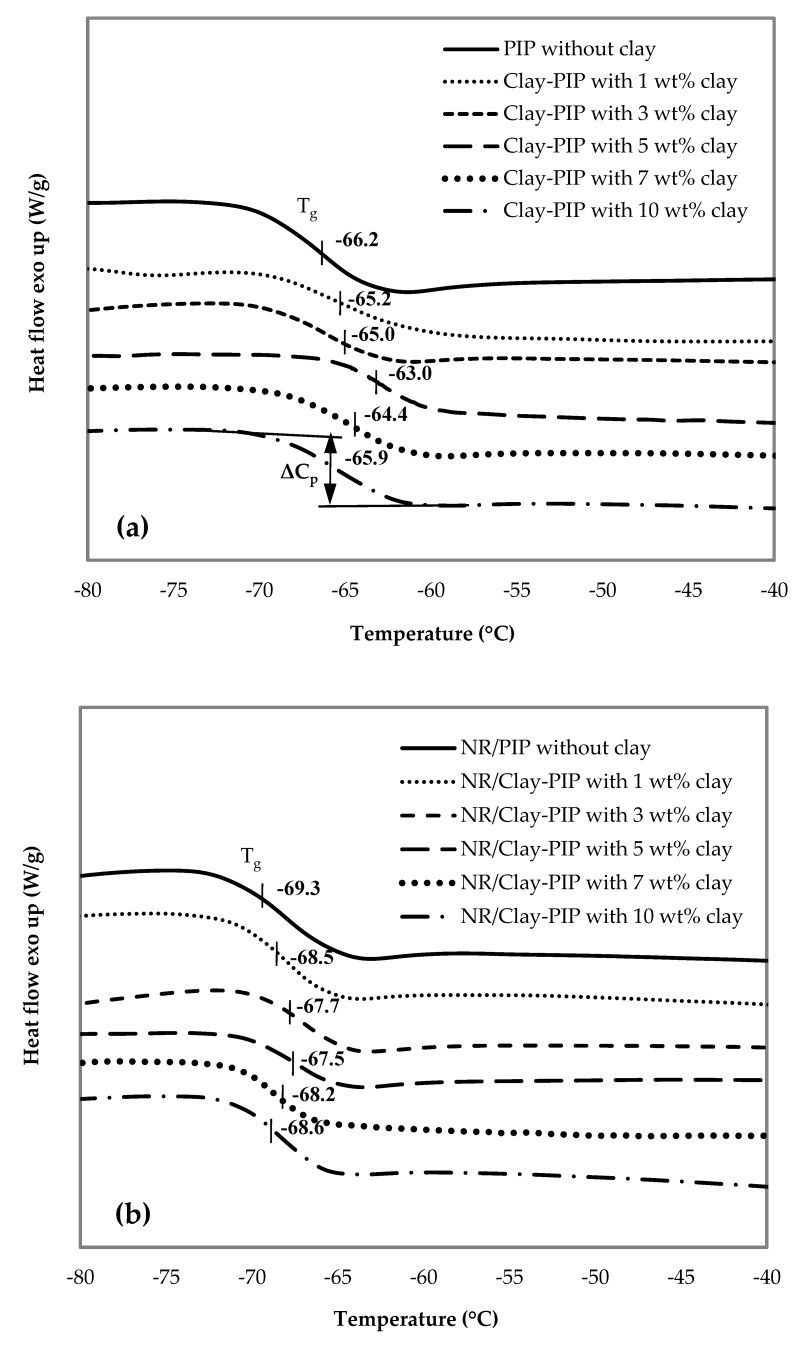
DSC thermograms of the (**a**) Clay-PIP and (**b**) NR/Clay-PIP composites containing various nanoclay contents. The Clay-PIP latexes were prepared using SFE technique. The clay contents are relative to the monomer amount.

Figure 7 shows DSC thermograms of the Clay-PIP solids with variable clay content. The variation of nanoclay amount in the modified isoprene latexes causes the T_g_ to shift to the highest values at the same wt% that gives the optimal mechanical properties (Figure 6). Adding the nanoclay at an optimum amount, i.e., 5 wt% rel. to the monomer content, increases the T_g_ of the composite to the maximum value, i.e., −63.0 °C, while the modified latexes without and with nanoclay at 1 and 3 wt% give T_g_ values of −66.2, −65.2, and −65.0 °C, respectively. Based on the concept of immobilized polymer as mentioned earlier, this result suggests the generation of a greater extent of immobilized polymer structure in the rubber composites. As a consequence, better reinforcement can be achieved when using the Clay-PIP modified with an optimum quantity of the nanoclay, i.e., 5 wt% relative to the monomer content. The modified isoprene latexes with a higher amount of nanoclay than the optimum level (>5 wt%), i.e., 7 and 10 wt% relative to the monomer content, give a drop in the glass transition temperatures. This is attributed to these amounts of nanoclay used are excessive, resulting in aggregation and poor dispersion of nanoclay in the polymer matrix, which leads to more inhomogeneity of the composites as well as lessened immobilized polymer constituent, consistent with the XRD results (Figure 5).

Another indicator used to determine the extent of the bound polymer layer at the interface between nanoclay and polymer matrix is the heat capacity (ΔC_p_) caused by molecular relaxation at the T_g_ transition region. The polymer molecules which interact with filler particles have less heat capacity throughout the molecular relaxation process, i.e., from on-set to end-set of the transition, since they are more restricted in mobility. The ΔC_p_ values of Clay-PIP with nanoclay of 1, 3, and 5 wt% are 0.25, 0.27, and 0.20 W/g, respectively, less than the value without nanoclay, i.e., 0.36 W/g. The composites with 7 and 10 wt% nanoclay relative to the monomer content have a higher ΔC_p_ value, i.e., 0.22 and 0.31 W/g, respectively, compared to the composite with 5 wt% nanoclay. The result of the ΔC_p_ measurement thus shows a good agreement with the T_g_ of the Clay-PIP composites.

**Figure 8:**
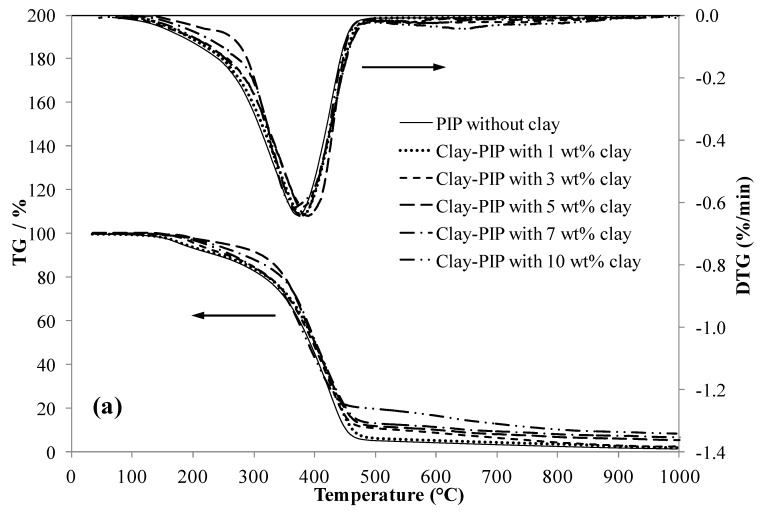

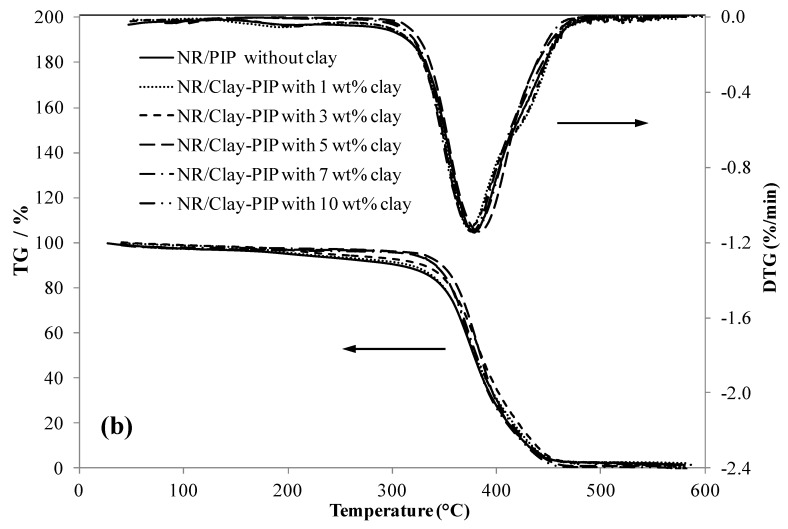
Thermogravimetric analysis (TGA) and DTG thermograms of the (**a**) Clay-PIP and (**b**) NR/Clay-PIP composites containing various nanoclay contents. The Clay-PIP latexes were prepared using SFE technique. The clay contents are relative to the monomer amount.

Another method to verify the extent of immobilized polymer structure of polymeric composites is to analyze their decomposition temperature (T_d_) since this bound polymer layer behaves as a rigid segment like filler particles [53], increasing stability toward decomposition at high temperatures. In other words, adding an appropriate amount of nanoclay results in a good heat resistance of NR/Clay-PIP composites. It can clearly be seen on the TGA and DTG curves that, in the temperature range of 200–350 °C, the curves are more stable when the composites contain a higher nanoclay content. The results shown in Figure 8 and Table 5 correspond well with the results of glass transition temperature (Figure 7). It reveals that the Clay-PIP composites containing nanoclay from 1–5 wt% relative to monomer content have an increase in the T_d_. The Clay-PIP solids with 7–10 wt% nanoclay have a gradual reduction in the T_d_ compared to the optimum one containing 5 wt% of nanoclay. This trend in Td is the same as seen in the tensile properties, morphology, XRD, and DSC results and can be ascribed to the same cause.

The 5 wt% nanoclay used to modify the polyisoprene latex gives the optimum mechanical properties of the NR thin films because this amount provides the maximized reinforcing power because of an optimum level of in-rubber structure or immobilized polymer as well as an appropriate degree of filler distribution in the elastomeric matrix. 

**Figure 9:**
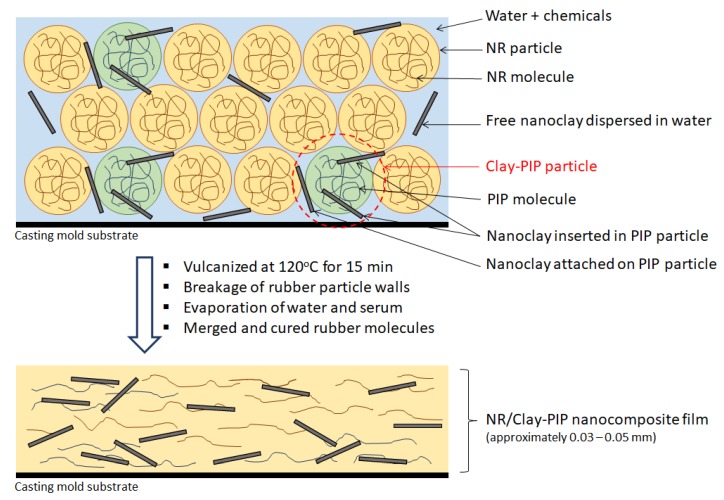
Film formation of the NR/Clay-PIP nanocomposite.

Figure 9 demonstrates how the NR/Clay-PIP latex can form a nanocomposite film. According to the actual values of nanoclay content in the dried form of Clay-PIP and NR/Clay-PIP film nanocomposites (Table 5), nanoclay contents in the composites increases with increasing the nanoclay used to modify the PIP latexes. Based on the information of this organo-nanoclay from the supplier, it contains the modifier “dimethyl, dehydrogenated tallow, quaternary ammonium” at about 40 wt% [54]. Therefore, the actual value of the nanoclay in the Clay-PIP with 1 wt% nanoclay relative to the monomer content (Table 5) is approximately 0.6 wt%, consistent with the TGA results. Applying the Clay-PIPs in the NR thin films, the actual amounts of nanoclay in the film composites appear to be very low. However, the reinforcing power is considerable, especially in the case of the Clay-PIP containing 5 wt% nanoclay relative to the monomer content. It is not only due to the greatest extent of immobilized polymer in this composite, but also due to an intrinsic character of the Clay-PIPs as reported in our previous work [43]. Polyisoprene synthesized in the present work consists of about 63 mol% trans molecular configuration which exhibits rather rigid compared to the cis-structure of natural rubber: see Figure 6. Therefore, this nanoclay-modified polyisoprene can increase the modulus of the NR film composites, as clearly observed from the stress–strain curves in Figure 6, contributing to improved reinforcement efficiency. 

## 4. Conclusions

Polyisoprene latexes were modified with organo-nanoclay during emulsion polymerization using batch and starve-fed methods. Starve-fed (SFE) and batch (BE) emulsion techniques have similar gel and coagulation contents. However, the SFE provides a better product conversion compared to the BE technique, i.e., approximately 5 wt% higher, since the SFE generates a higher particle concentration, leading to more loci of the reaction and thus the better polymerization efficiency. Increasing organo-nanoclay content results in a larger average polymer particle size as well as a broader particle size distribution because of the competition between the organo-clay and PIP particles for surfactant, leading to a lower particle number. TEM results were consistent with the particle size analysis and showed that the nanoclay particles are inserted in and/or attached on the polyisoprene particles suspended in latexes. XRD results show that the addition of nanoclay from 1 to 5 wt% relative to the isoprene monomer content increases the d-spacing of nanoclay structure. At 10 wt% of nanoclay, the d-spacing is reduced, presumably due to reagglomeration. The obtained nanoclay-modified polyisoprene latex with varied nanoclay contents were used as a reinforcing agent for natural rubber thin films. The films filled with the modified isoprene latexes containing organo-nanoclay from 1 to 5 wt% relative to the monomer content show an increase in tensile strength and modulus with a slightly reduced elongation at break, attributed to an increasing amount of immobilized polymer segments at the clay surfaces. However, adding the modified isoprene latexes containing nanoclay at 7 and 10 wt% leads to reduced strength and modulus of NR thin films, while maintaining elongation at break. DSC and TGA analyses confirm that the reinforcement mechanism of this nanoclay-modified polyisoprene to the natural rubber thin films occurs mainly through the contribution of immobilized polymer because of the inclusion of nanoclay.

## Figures and Tables

**Table 1:** Synthesizing recipes of polyisoprene (PIP) and Clay-PIP Latexes containing various nanoclay contents. Two techniques were employed: batch (BE) and starve-fed (SFE) emulsion polymerizations. Sodium dodecyl sulfate (SDS) at 0.25 wt%, *n*-DM at 0.05 wt%, NaHCO_3_ at 0.15 wt%, and KPS at 0.15 wt% relative to the total weight of an obtainable latex (i.e., 100 wt% = 200 g) were used.

Latexes	System	Nanoclay (wt%)
Clay-PIP (BE) with 5 wt% clay	BE	0.5
PIP without clay	SFE	-
Clay-PIP with 1 wt% clay	SFE	0.1
Clay-PIP with 3 wt% clay	SFE	0.3
Clay-PIP with 5 wt% clay	SFE	0.5
Clay-PIP with 7 wt% clay	SFE	0.7
Clay-PIP with 10 wt% clay	SFE	1.0

**Table 2:** Latex compound formulations used for preparing the natural rubber thin films.

Ingredients	Dry Weight (phr)
Rubber latex ^a^	100
KOH	0.5
K-Laurate	0.5
Sulfur	1.4
ZDEC	0.9
ZMBT	0.5
Antioxidant	0.9
ZnO	2.7

^a^ Rubber latex was based on the blend of NR and Clay-PIP latexes.

**Table 3:** Basic properties of the Clay-PIP latexes with different amounts of nanoclay prepared using batch (BE) and starve-fed (SFE) emulsion polymerization techniques.

Latexes	Conversion (wt%)	Gelation (wt%)	Coagulation (wt%)
Clay-PIP (BE) with 5 wt% clay	65.4 ± 5.9	21.7 ± 4.3	0.8 ± 0.2
PIP without clay	91.7 ± 8.2	22.5 ± 5.2	0.3 ± 0.0
Clay-PIP with 1 wt% clay	78.3 ± 7.1	21.8 ± 4.8	0.3 ± 0.1
Clay-PIP with 3 wt% clay	77.8 ± 6.3	21.1 ± 4.0	0.4 ± 0.1
Clay-PIP with 5 wt% clay	69.9 ± 5.1	18.9 ± 3.5	0.5 ± 0.2
Clay-PIP with 7 wt% clay	67.3 ± 5.8	18.1 ± 3.1	0.7 ± 0.2
Clay-PIP with 10 wt% clay	63.4 ± 6.3	19.2 ± 3.9	1.5 ± 0.4

**Table 4:** Polymer particle size and polymer particle number of Clay-PIP latexes with varied nanoclay contents prepared using batch (BE) and starve-fed (SFE) emulsion polymerization techniques.

Latexes	Particle Size (µm)	Particle Dispersity	Particle Number (L^−1^)
Clay-PIP (BE) with 5 wt% clay	1.40	0.43	5.2 × 10^13^
Clay-PIP without clay	1.01	0.10	15.5 × 10^13^
Clay-PIP with 1 wt% clay	1.05	0.19	14.0 × 10^13^
Clay-PIP with 3 wt% clay	1.11	0.36	11.7 × 10^13^
Clay-PIP with 5 wt% clay	1.14	0.35	9.8 × 10^13^
Clay-PIP with 7 wt% clay	1.24	0.40	7.3 × 10^13^
Clay-PIP with 10 wt% clay	1.46	0.62	4.2 × 10^13^

**Table 5:** Thermal properties of Clay-PIP and NR/Clay-PIP composites with different amounts of nanoclay. The Clay-PIP were prepared using starve-fed (SFE) emulsion polymerization technique.

Composites	T_g_	ΔC_p_	T_d_	Actual Nanoclay
	(°C)	(W/g)	(°C)	Content (wt%) ^a^
PIP without clay	−66.2	0.31	374	0.00
Clay-PIP with 1 wt% clay	−65.2	0.25	377	0.60
Clay-PIP with 3 wt% clay	−65.0	0.27	378	1.70
Clay-PIP with 5 wt% clay	−63.0	0.20	382	4.20
Clay-PIP with 7 wt% clay	−64.4	0.22	380	5.90
Clay-PIP with 10 wt% clay	−65.9	0.31	375	7.60
NR/PIP without clay	−69.3	0.38	378	0.00
NR/Clay-PIP with 1 wt% clay	−68.5	0.36	371	0.01
NR/Clay-PIP with 3 wt% clay	−67.7	0.31	376	0.04
NR/Clay-PIP with 5 wt% clay	−67.5	0.31	383	0.08
NR/Clay-PIP with 7 wt% clay	−68.2	0.34	369	0.12
NR/Clay-PIP with 10 wt% clay	−68.6	0.36	371	0.15

^a^ The amount of nanoclay in the composites was calculated based on the TGA results. A residual at 1000 °C was considered as the total weight of non-decomposed carbon (ash) and filler. The ash contents were corrected for the ones containing Clay-PIPs by taking into account the residual weight of unfilled PIP and NR/PIP in order to obtain only the amount of nanoclay.
